# Synthesis and retarder mechanism study of a novel amphoteric composite high temperature-resistant retarder for oil well cement

**DOI:** 10.1039/c8ra01139g

**Published:** 2018-04-19

**Authors:** Peng Zhigang, Zhang Jian, Feng Qian, Zou Changjun, Zheng Yong, Zhang Bojian, Huo Jinhua

**Affiliations:** The School of Chemistry and Chemical Engineering, Southwest Petroleum University 610500 Sichuan China 529468317@qq.com 116004373@qq.com

## Abstract

An amphoteric composite polymer (hereinafter referred to as PAADM) as a high temperature-resistant cement retarder was prepared by *in situ* intercalated polymerization method with 2-crylamido-2-methylpropanesulfonic acid (AMPS), acrylic acid (AA) and two diallyl dimethyl ammonium chloride (DMDAAC) as monomers, and modified montmorillonite as an active polymerization filler. The synthetic composite polymer was characterized by Fourier transform infrared spectroscopy (FT-IR), H nuclear magnetic resonance (H-NMR), X-ray diffraction (XRD) and thermogravimetric analysis (TGA). The results of the aforementioned characterization showed that the synthesized copolymer (PAADM) has an intercalation/exfoliation composite structure and excellent thermal stability. Performance evaluation evidenced that the cement slurry containing PAADM has good retarding property in the range of 120–200 °C, and demonstrated the rapid development of compressive strength under high temperature and low temperature conditions, this property could guarantee that the retarder PAADM could be applied to the construction of deep wells and long interval wells. Moreover, the retarding mechanism of PAADM was studied through calcium binding capacity, adsorption amount, zeta potential, XRD and SEM analysis, and it was found that “adsorption deposition” and “calcium complexation” should be responsible for this retarder delaying the hydration process of cement grains.

## Abstract 2

An amphoteric composite polymer (hereinafter referred to as PAADM) as high temperature-resistant cement retarder was prepared by *in situ* intercalated polymerization method with AMPS, AA and DMDAAC as monomers, and modified montmorillonite as an active polymerization filler.

## Graphical abstract

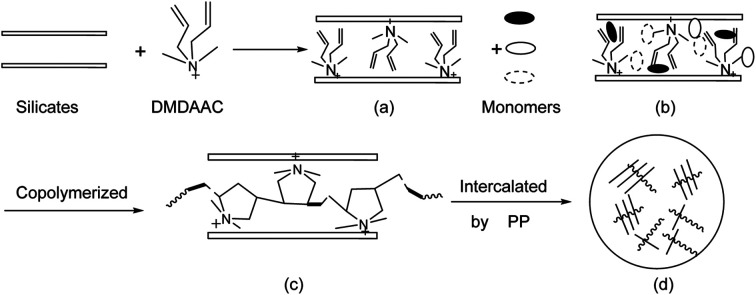

## Introduction

In order to isolate the oil, gas and water layers to ensure the production and reparation of oil wells, the cementing operation is necessary in every oil and gas well.^1^ The cementing of oil and gas wells also is an irreparable and most high-risk operation in the construction of a well bore.^2^ During cementing operations, it is essential for cement slurry to remain pump-able during introduction into the subterranean formation and until the slurry is situated in the desired portion of the well to be cemented.^3^ Therefore, a cement retarder is introduced into cement slurry to control the setting process and retain it in flowable and pumpable state during the whole cementing operation. Retarders, which are one of three main additives for cement slurry, are obviously included to prolong the setting time or thickening time of the cement slurry due to hindering the hydration of cement clinkers and they provide the sufficient pumpability that cement slurries require to be placed in a desired portion of the well.^4^

Currently, a wide variety of retarders are applied to oil and gas well cementing to control the pumpability and the safety of cementing operation. The traditional retarders are usually composed of lignosulfonate, cellulose, tannin, tartaric acid, saccharide, boric acid and organic phosphine.^5^ The aforementioned retarders have good retarding effect in low and middle temperature environment, but must be compounded with others retarder before being applied to high temperature environment. Since 1990s, various polymeric retarders had gradually become the preferred retarder and been extensively used in cementing operations for several decades, especially in deep well and ultra deep wells, by virtue of their uniformity, thermal and salt stability. Consequently, the polymeric retarders have attracted great interest of domestic and abroad researchers in petroleum industry. A high temperature polymeric retarder adapted to 180 °C was prepared by Guo *et al.* using AMPS, IA and an unsaturated cationic monomer.^6^ Tiemeyer and Plank synthesized another high temperature retarder comprising of 2-acrylamido-2-methyl propane sulfonic acid (AMPS) and itaconic acid (IA), the effective application temperature of which is increased to 200 °C.^7^ Some polymeric retarders with better high-temperature performance were synthesized by Dong *et al.*, Khaild *et al.* and Gosavi *et al.*^8–10^ using AMPS or SSS as the functional main monomers. Most of this synthetic polymeric retarders can effectively prolong the thickening time of cement slurry at high temperature conditions, however, there were two main defects that prevent them from being widely applied in the filed. One is the “super-retardation” phenomenon of cement slurry due to the sensitivity of retarders to temperature, this phenomenon directly leads to a significant decrease in the compressive strength of cured cement stone, especially in the low temperature environment, the compressive strength (<3.5 Mpa/24 h) is not enough to support the casing string and ensure the further drilling.^11^ And the other one is still the poor thermal stability. Though the thickening time or setting time is effectively prolonged, the thickening curve of cement slurry containing retarders shows a obvious bulging phenomenon attributed to the decomposition of function groups and molecule chains under high temperature conditions.^12^

To the best of our knowledge, at presently, there are very few literature about the retarders meeting the performance requirements of both retardation and compressive strength at a wide temperature range. Therefore, in this paper, a new type of amphoteric composite retarder (PAADM) was prepared by *in situ* intercalated polymerization method using 2-acrylamido-2-methylpropanesulfonic acid (AMPS), acrylic acid (AA), two diallyl dimethyl ammonium chloride (DMDAAC) as monomers, modified montmorillonite as active polymerization filler. Then, the structure and thermal stability of the retarder PAADM were characterized by Fourier transform infrared spectroscopy (FT-IR), H nuclear magnetic resonance (H-NMR), X-ray diffraction (XRD) and thermogravimetric analysis (TGA), and the retardation performance, effects of the retarder on the compressive strength of setting cement stones and other comprehensive properties of setting cement slurry were evaluated respectively. Furthermore, the working mechanisms of the retarder PAADM was studied and analyzed, which not only has important practical significance to deal with the actual requirements in oil field cementing engineering, but also provides a theoretical reference for the development of similar oil field chemicals.

## Experimental

### Materials

2-Acrylamido-2-methyl propane sulfonic acid (AMPS) and diallyl dimethyl ammonium chloride (DMDAAC) were received from Jintong Letai Chemical Product Co., Ltd (Beijing, China). Acrylic acid (AA) and potassium persulfate (KPS) were acquired from Kelong Chemical plant (Chengdu, China). Sodium montmorillonite (Na-MMT) (the cation exchange capacity (CEC) is 116 meq/100 g) and silicon powder (the content of SiO_2_ more than 98%) were purchased from Anxian Huaxi Mineral Powder Co., Ltd (Chengdu, China). The Class G MSR oil well cement was produced by Sichuan Jiahua cement plant. The Blaine value of which is 318 m^2^ kg^−1^ and the phase composition was determined by means of X-ray diffraction (XRD)and calculated according to the Bogue equation.^13^ The chemical and mineralogical compositions of the cement are indicated in Table 1. The dispersant (SXY), fluid loss reducer (SZ1-2) and defoamer (D50) were all obtained from Sichuan southwest Ten Jinniu Oil Technology Co., Ltd (Chengdu, China). Deionized water (DI water) and tap water are used separately in the polymerization process of the retarder PAADM and the preparation of cement slurries. All of the aforementioned chemicals were analytical grade.

**Table 1:** Chemical and mineralogical compositions of the Class G MSR oil well cement

Oxide	wt%	Phase	wt%
CaO	59.80	C_3_S	49.30
SiO_2_	22.40	C_2_S	24.70
Fe_2_O_3_	7.60	C_3_A	6.80
Al_2_O_3_	2.70	C_4_AF	11.0
SO_3_	2.40	CaSO_4_	4.10
MgO	2.60	Others	3.80
K_2_O	0.60		
Na_2_O	0.20		
Others	0.70		

### Synthesis of amphoteric composite retarder PAADM

#### Synthesis of organically modified sodium montmorillonite (O-MMT)

24.72 g diallyl dimethyl ammonium chloride (DMDAAC) were gradually added to 400.00 g sodium montmorillonite suspension with a mass concentration of 5.00% which had been prehydrated for 24 hours, and the resulting suspensions were ultrasonically dispersed for 6 hours at room temperature, then the organically modified sodium montmorillonite suspension by diallyl dimethyl ammonium chloride were obtained. The organically modified sodium montmorillonite were filtered and washed by DI water until no further formation of AgCl was detected after addition of 0.1 N AgNO_3_ to the washing water. The treated sodium montmorillonite was dried in vacuum at 100 °C for 24 hours and grounded into powders, finally, the organically modified sodium montmorillonite (O-MMT) was prepared.

#### Synthesis of amphoteric composite retarder PAADM

**Fig. 1:**
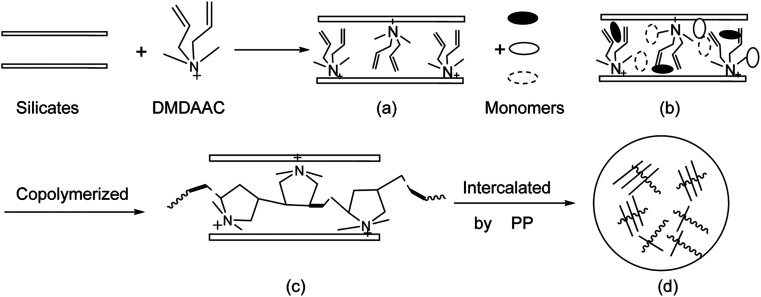
Schematic illustration of the formation process of amphoteric composite retarder PAADM.

**Fig. 2:**
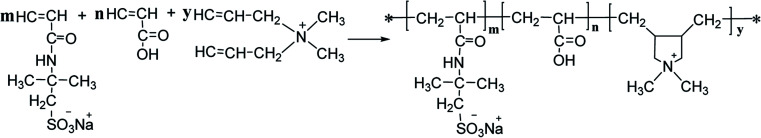
Schematic representation for the polymerization equation of amphoteric composite retarder PAADM.

The amphoteric composite retarder (PAADM) were synthesized by *in situ* free-radical intercalated polymerization of AMPS/AA/DMDAAC in the presence of modified sodium montmorillonite (O-MMT). Firstly, the aforementioned montmorillonite (O-MMT) (2.50 g)was dispersed in 70 g DI water with ultrasonic vibration for 3.0 hours at room temperature. Subsequently, 12.50 g AMPS, 8.30 g AA and 2.20 g DMDAAC were added to the montmorillonite (O-MMT) suspension and placed into the 250 mL four-nacked flask with a stirring rate (220 ± 10 rpm) and nitrogen protection. The pH value of mixed suspension was adjusted to 6–7 with prepared sodium hydroxide solution, and the mixed solution continued to stir for 0.5 hour to ensure that the organic montmorillonite (O-MMT)were fully saturated and swelled by monomers mentioned above. After completion of the above operation, 5.0 g initiator solution with a mass fraction of 3% were added by drop to complete the polymerization over the periods of 6.0 hours at 65 °C. After the products were cooled to room temperature, orange red and viscous polymer solution were obtained. The obtained copolymer solutions were extracted by enough ethanol repeating for three times and dissolved in DI water again for dialysis treatment to remove residual monomers and ions. After drying and crushing, the orange red powder of retarder PAADM was prepared finally. The formation process and polymerization equation are shown in Fig. 1 and 2 respectively.

#### Characterization of amphoteric composite retarder PAADM

##### Chemical structures analysis

The chemical structures of the purified retarder PAADM were characterized by a FTIR (Nicolet 6700 FTIR, Thermo Fisher Scientific, USA) instrument with a resolution of 4 cm^−1^ and a NMR (Bruker AvanceIII 400 NMR Bruker BioSpin, Switzerland) instrument by dissolving the samples in deuterium oxide (D2O). The purified PAADM powers were homogeneously mixed with KBr salt and completely dried in a vacuum oven at 80 °C, the mixture was pressed into a tablet and then scanned in the transmission mode from 400 to 4000 cm^−1^at a room temperature of 25 °C.

##### XRD analysis

The X-ray diffraction (XRD) patterns of modified montmorillonite (O-MMT) and composite retarder PAADM were performed through a X'Pert MPDPRO X-ray diffractometer (Bruker, Germany) at a rate of 0.02° s^−1^ in the 2*θ* range from 3° to 15°. The interlayer spacing of modified montmorillonite (O-MMT) and its in retarder PAADM are calculated from Bragg's law: 2*d* sin *θ* = *nλ*. Where *λ* is the wavelength of the incident beam (*λ*(CuKα) = 0.1542 nm), 2*θ* is the angle between incident and scattered X-ray wavevectors, and *n* is the interference order (*n* = 1).^14,15^

##### TGA analysis

Thermogravimetric analysis (TGA) of the retarder PAADM was carried out using a NetzschSTA449F3 differential thermal analyzer (NetzschSelb, Germany). The PAADM samples of 10.13 mg each in an alumina crucible were used for the TGA test with a heating rate of 5 °C min^−1^ at atmospheric pressure, and the temperature range was from 40 to 500 °C.

### Performance evaluation of cement slurry and characterization of setting harden cement stones

The preparation and performance evaluation of the set cement slurry with retarder are based on API Recommended Practice 10B “Recommended Practice for Testing Well Cements”^16^ and the oil and gas industry standard SY/T5504.1-2005 “Evaluation Method for Well Cement Additives: Part 1, Retarder”^17^ respectively.

#### Thickening time

Thickening time of the cement slurry with retarder was determined using a OWC-9040F high pressure and high temperature consistometer (JinOke, China) after completion of the set cement slurry preparation according to the standard of API Recommended Practice 10B. The slurry was poured into the steel vessel and rotated at a speed of 150 ± 15 rpm. When the consistency of cement slurry reaches 70Bc, the experiment will be stopped.

#### Compressive strength

To determine the compressive strength, the set cement cubes (50 mm × 50 mm × 50 mm) were removed from the molds and investigated using NYL-300L pressure testing machine (Building Materials Technology Equipment Corporation, China), where increasing force was exerted on each cube until failure, in compliance with API Recommended Practice 10B for oil well cement testing. The test results of compressive strength were reported as an average of three parallel samples.

#### Adsorption amount

The amount of retarders adsorbed on cement were determined according to the depletion method, the amount of retarders retained *via* adsorption on cement was calculated from the difference between the polymer concentration added to the cement slurry and the amount of non-adsorbed polymer found in solution. A series of retarders solution with different concentration were prepared in advance. Then, the solution (20 g) and cement (1.0 g) were mixed and stirred for 120 minutes to achieve adsorption equilibrium. After that, the suspension was separated by centrifuging it at 5000 rpm for 15 minutes. The organic carbon content of upper supernatant was measured by TOC analyzer (Multi N/C2100, Germany) to calculate the adsorption amounts of retarders, measurements were generally repeated three times to obtain the average results, with the results within 5% deviation. The adsorption amount (mg g^−1^-cement) of retarders can be calculated from the residual concentration of polymer as follows:Adsorption amount (mg g^−1^) = *V*(*C*_0_ − *C*)/*m*where *C*_0_ = initial concentration (g L^−1^)of retarders before adsorption; *C* = residual concentration (g L^−1^) after adsorption; *V* = volume of the solution (mL); and *m* = mass of the cement (g).

#### SEM analysis

The mineral phase microstructure of cement stones containing different dosages of retarder under setting test conditions (cured for 24 hours at 90 °C respectively by water bath) were characterized by scanning electron microscopy (Quanta450, FEI, American). Prior to the SEM examination, the specimens must be shaped and dried in a drying oven, gold coating for specimens is also required.

#### XRD analysis

The mineral phase compositions of the cement stones containing different dosages of retarder under setting test conditions (cured for 24 hours at 90 °C respectively by water bath) were characterized using a X'Pert MPDPRO X-ray diffractometer (Bruker, Germany) at a rate of 0.02° s^−1^ in the 2*θ* range from 5° to 70°. The specimens need to be milled into powder (<32 μm) before the X-ray diffraction measurement.

## Results and discussion

### The chemical structure of retarder PAADM

**Fig. 3:**
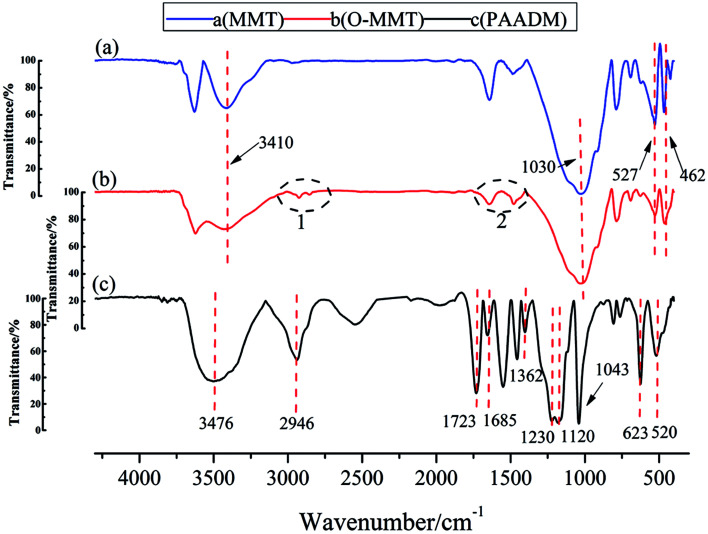
Infrared spectra of montmorillonite, modified montmorillonite and PAADM.

**Fig. 4:**
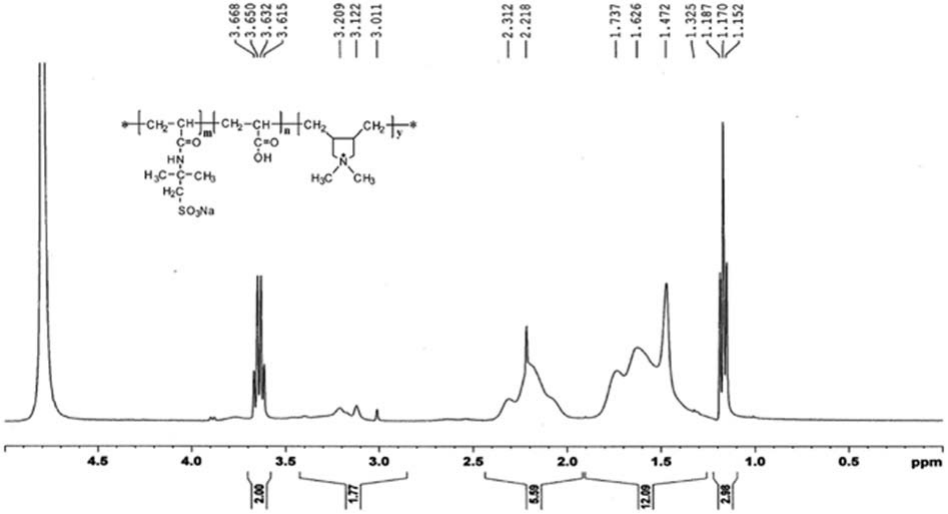
H-NMR spectrum of PAADM, measured in D_2_O.

**Fig. 5:**
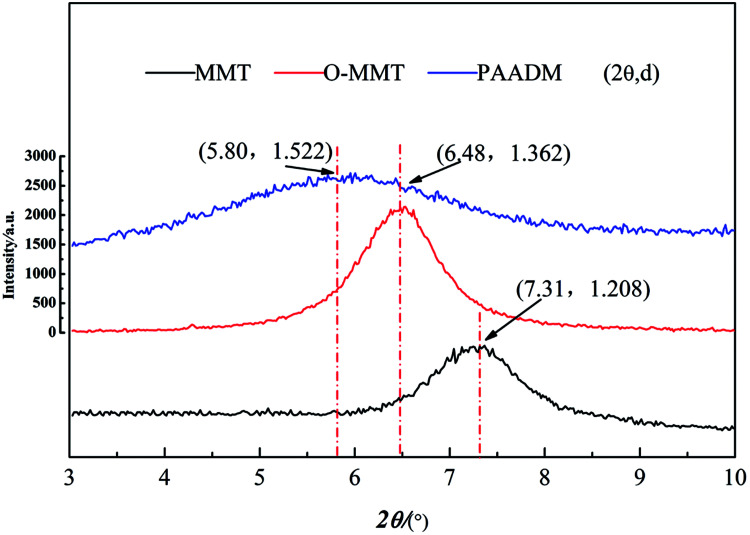
XRD spectra of montmorillonite, modified montmorillonite and PAADM.

To illustrate the molecular structure of the composite polymer, the FTIR spectrum, H-NMR spectrum and XRD spectrum of montmorillonite, modified montmorillonite (O-MMT) and the obtained polymer PAADM are showed in Fig. 3–5 respectively.

As seen from Fig. 3, in the spectrum of MMT (a) and O-MMT (b), the peaks at 3410 cm^−1^ and 1030 cm^−1^ are respectively assigned to the stretching vibration of O–H and Si–O in MMT. The peaks presented at 527 cm^−1^ and 462 cm^−1^ are respectively contributed by the bending vibration of Al–O and Si–O in MMT. Comparing the two infrared spectra mentioned above, there are two groups of peaks at 2800–3000 cm^−1^ appeared in the infrared spectra of OMMT (black circles 1 shown in the Fig. 3(b)), these peaks belong to the stretching vibration of methyl or methylene. Futhermore, there are two groups of peaks at 1680–1600 cm^−1^ appeared in the infrared spectra of OMMT (black circles 2 shown in the Fig. 3(b)), these peaks belong to the stretching vibration of C=C in DMDAAC, the appearance of these peaks indicate that the montmorillonite successfully modified by diallyl dimethyl ammonium chloride (DMDAAC). In the spectrum of PAADM, the characteristic peaks at 3476 cm^−1^ is contributed by the stretching vibration of O–H in carboxyl group of acrylic acid (AA), and 2946 cm^−1^ is the characteristic absorption band of methyl (–CH_3_). The peaks at 1723 cm^−1^ and 1685 cm^−1^ are respectively allocated to the stretching vibration of C=O in AA and 2-acrylamido-2-methyl propane sulfonic acid (AMPS). The peaks occurred at 1230 cm^−1^ and 1043 cm^−1^ are attributed to the vibration of the sulfonate group in AMPS. The peak presented at 1362 cm^−1^ is assigned to the vibration of C–N in DMDAAC. The absorption peaks at 1120, 623 and 520 cm^−1^ are not the characteristic peaks of the polymers, but originate from the stretching vibration of Si–O and the flexural vibration of Al–O and Si–O in MMT. In addition, it can be seen from the H-NMR spectrum (Fig. 4)that the strongest peak at *δ* = 4.66 ppm is the vibration peak of the solvent D_2_O. *δ* = 3.63 ppm can be assigned to the methylene connected with the sulfonic group in AMPS, *δ* = 1.15–1.19 ppm are contributed by the methyl resented in side chain of AMPS. *δ* = 3.21, 3.12 and 3.01 ppm were introduced by methyl connected with N^+^ in DMDAAC, the methylene connected with N^+^ in DMDAAC is responsible for the peak at *δ* = 3.67 ppm. Moreover, *δ* = 1.32, 1.47 ppm belong to the proton peak of methylene present in the backbone of the polymer. All of these information clearly evidence that the three monomers and modified montmorillonite have all involved in the polymerization.

Futhermore, it can be seen from the XRD spectrum (Fig. 5) that a strong diffraction peak at 2*θ* = 7.3° of MMT corresponding to the layered structure (spacing between clay sheets *d* = 1.208 nm) moved to the left with the modification or polymerization. The characteristic diffraction peaks of O-MMT and PAADM are respectively allocated to 2*θ* = 6.48° and 5.80°, the corresponding interlayer spacing increases to 1.362 nm and 1.522 nm. These XRD results indicate that the MMT layers have been intercalated successfully by DMDAAC and polymers, the PAADM is a kind of composite material with intercalation structure. Combined with the FTIR, H-NMR and XRD spectra in Fig. 3–5, it can be concluded that the three monomers and modified montmorillonite have all taken part in the polymerization, and the amphoteric composite polymer (PAADM) with intercalation structure is successfully synthesized.

### Thermal stability of retarder PAADM

**Fig. 6:**
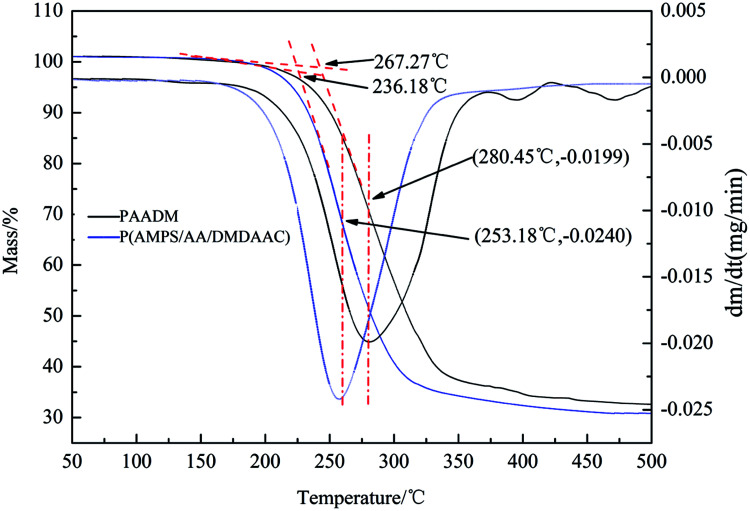
The TGA and DTG curves of the retarder PAADM.

As a high temperature oil well cement retarder, PAADM should have good temperature resistance to meet the requirements of cementing operation, the weight loss curve and weight-loss rate curve of PAADM are shown in Fig. 6.

The weight loss curve and weight-loss rate curve of PAADM are shown in Fig. 6. The results shown that the initial thermal decomposition temperature of P(AMPS/AA/DMDAAC) is 236.18 °C, the rate of weight-loss reaches the fastest at the temperature of 253.18 °C, where the corresponding weight loss rate and total thermogravimetric mass loss are respectively 0.0240 mg min^−1^ and 32%, the total thermal weight loss is about 65% within the range of 350 °C. While the initial thermal decomposition temperature of PAADM with O-MMT increases to 267.27 °C, the temperature with the fastest weight loss ratio is also up to 356.35 °C, where the corresponding weight loss rate and total thermogravimetric mass loss separately reduce to 0.0199 mg min^−1^ and 27%, the total thermogravimetric mass loss reduces to 60% within the range of 350 °C. Therefore, the results of TGA-DTG analysis indicate that the PAADM possesses a stable structure and an excellent thermal resistance. The following two aspects should be responsible for the excellent thermal resistance: the inorganic material montmorillonite (MMT) gives itself rigidity and thermal stability to the polymers (PAADM). The polymer is intercalated between the MMT layers, so the amide and carboxyl groups in PAADM molecules were protected by the layered structure, which effectively shields the thermal decomposition of polymer molecules.

### Temperature resistance retarding property of PAADM

**Fig. 7:**
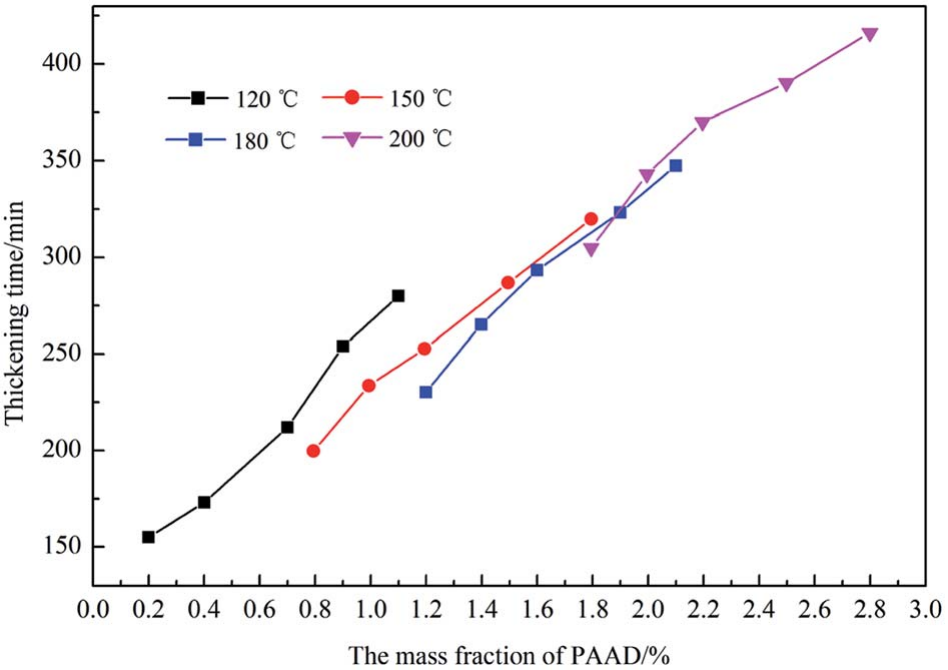
Effect of PAADM dosage on thickening time at different temperatures.

**Fig. 8:**
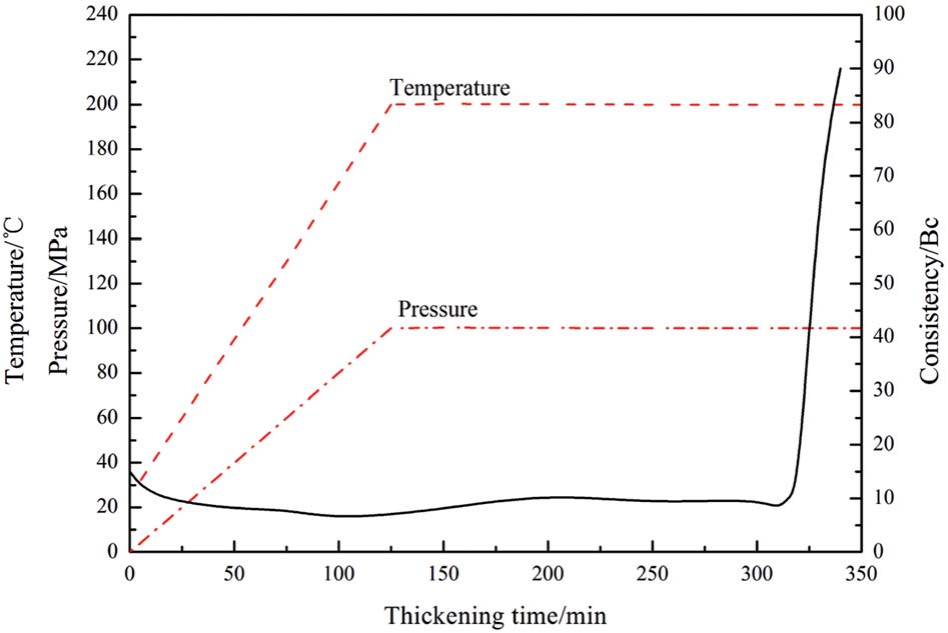
Consistency curve of cement slurry with 2.0% PAADM at 200 °C.

Under high-temperature conditions, polymeric retarders usually have the tendency to suffer degradation of function groups and rupture of the molecule chain, which results in weakening of the ability to maintain the cement slurry pumpability. To quantify the high temperature retarding effectiveness of the retarder PAADM, dosages-temperatures-dependent thickening times of cement slurries containing PAADM were measured at temperature 120 °C up to 200 °C (Fig. 7). It was found from the Fig. 6 that PAADM could prolong the thickening time of cement slurry when the mass fraction of retarder increased at different temperatures. It also can be seen from the curve that the thickening time decreases as the temperature increases from 120 °C to 200 °C. There is a good linear relationship among the thickening times, the dosages and temperatures. From the results of Fig. 6, it is apparent that PAADM has good adjustability of thickening times and excellent property of retardation on cement hydration under high temperature and pressure. Fig. 8 is the thickening curves of cement slurry with 2.0% PAADM at 200 °C, 100 MPa. It reveals that the thickening time of cement slurry with 2.0% PAADM could up to 327 min, which fully meets the safety requirements of cementing construction. During the gradually increased of temperature and pressure, the thickening curve has a short transition time (less 15 min) from 40 Bc to 100 Bc and the initial consistency has been below 25 Bc. Lower initial consistency and shorter transition time meant that cement slurry has good pumpability and anti-gas channeling performance under dynamic conditions present in deep well. Furthermore, the thickening curve is smooth and stable, there are no abnormal phenomenon such as “bulging” and “walking steps”. It shown that the retarder PAADM could make cement slurry possess good retarding stability under high temperature-pressure conditions.

### The influences of PAADM on the compressive strength

**Fig. 9:**
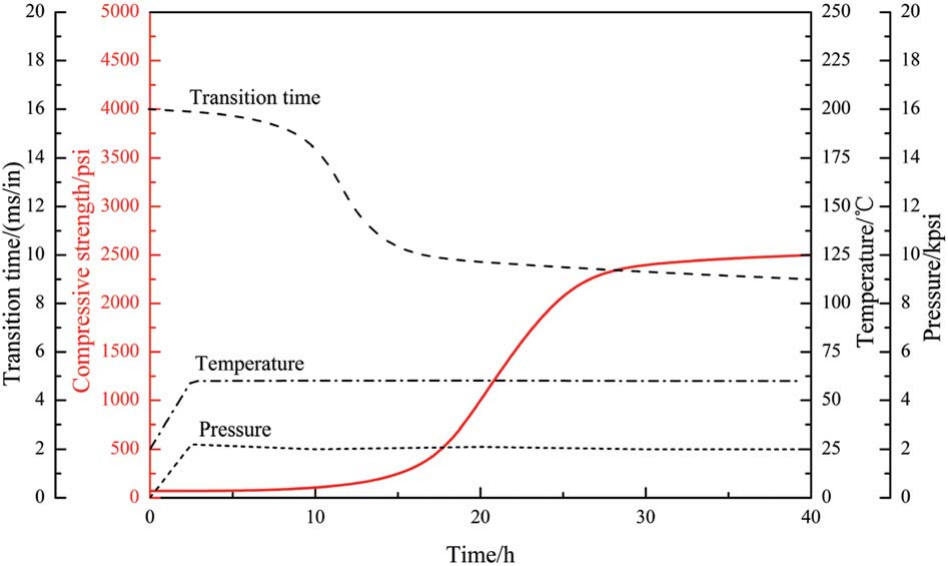
Compressive strength development curve of cement paste at 60 °C/20 MPa (1 in = 2.54 cm, 1 psi = 6894 kPa).

The conventional high temperature retarder often causes “super-retardation” phenomenon of cement slurry in the low temperature zone of long cementing interval of deep well, this phenomenon seriously inhibits the early-age compressive strength development of cement stone, which cannot meet the engineering requirements of secondary drilling and supporting the casing string. So it is vital for the oil-well cement slurry to develop a satisfactory compressive strength within 24 h, 48 h at different temperatures, especially at low temperature conditions in a high temperature–pressure deep well. Hence, the compressive strength of slurry with different dosages of PAADM was evaluated after curing at different temperatures and the results are presented in Table 2. The values indicate that the 24 hours compressive strength of set cements with different retarder dosages are more than 27.00 MPa at high temperature, which shown that the retarder can ensure the rapid development of cement compressive strength under high temperature environment. However, when cement stones are cured at low temperature, the development of compressive strength is affected at different degrees by the addition of PAADM, especially the 24 hours compressive strength. In detail, the 24 hours compressive strength of set cements with different retarder dosages decreases to more than 16.00 MPa and 14.00 MPa respectively at 90 °C and 60 °C, which is far larger than the criterion of the secondary drilling cement strength 3.5 MPa. With the increase of curing time, the 48 hours compressive strength increased to more than 23.00 MPa and 18.00 MPa respectively at 90 °C and 60 °C. The above phenomenon indicates that the adverse effect of retarder on cement compressive strength is gradually weakened with the prolongation of curing time, and the compressive strength can be maintained at more than 18.00 MPa under low temperature environment. The compressive strength development curve of the cement slurry system with 1.6% PAADM is shown in Fig. 9 under the condition of 60 °C and 20 MPa. It can be seen that the compressive strength of the cement stone began to develop rapidly at 15 h, and reached 14.02 MPa at 24 h. After 30 hours, the strength development slowed down to 17.25 MPa and maintained growth trend. Corresponding to the results of the thickening and compressive strength, it is shown that the retarder PAADM can meet the requirements of cementing pumpability and secondary drilling operations, moreover, it can effectively avoid the phenomenon of super-retardation at low temperature. Therefore, the retarder PAADM can be used not only in deep well cementing, but also applied to a long interval cementing.

**Table 2:** Effect of PAADM on compressive strength of cement stone at different temperatures

The dosage of PAADM/%	Curing temperature/°C	Compressive strength/MPa, 24 h	Compressive strength at different temperature difference/MPa			
			Top temperature 90 °C		Top temperature 60 °C	
			24h	48h	24h	48h
1.2	120	27.61	17.31	26.82	15.12	19.44
1.4	150	27.83	16.20	23.63	14.89	18.72
1.6	180	28.42	17.13	27.45	14.56	18.65
1.8	200	29.35	16.84	26.28	14.32	18.82

### Retardation mechanism of PAADM

To understand the retarding mechanisms of the retarder PAADM, the effect of Ca^2+^ concentration on the specific anionic charge density of PAADM was conducted firstly by charge titration using a cationic polyelectrolyte. Next, the adsorbed amount of retarder on cement particles and the zeta potential of cement slurries added with the retarder were measured. Finally, the effect of retarder on the hydration products and microstructures of set cement stone was investigated by SEM and XRD analysis.

#### Calcium binding capacity

**Fig. 10:**
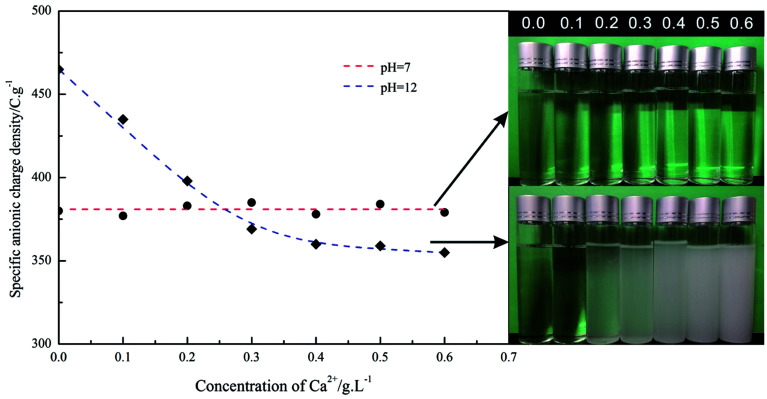
Effect of Ca^2+^ concentration on the specific anionic charge density of PAADM (*c*_polymer_ = 1.0 g L^−1^), measured at pH = 7 and 12 respectively.

The charge density change trend of PAADM with the concentration of calcium ions was tested in neutral (pH = 7) and alkaline (pH = 12, equivalent to the cement pore solution pH value) environment respectively. The results are displayed in Fig. 10. It is clearly seen that at pH = 7 the anionic charge density of PAADM is independent of the Ca^2+^ concentration and attains a value of −380 C g^−1^, the addition of Ca^2+^ don't result in precipitation in the retarder solution, and the solution has been kept in a transparent state. The calcium complex capacity difference of carboxylic groups and sulfonate groups and the electrostatic repulsion between calcium ions and anionic N^+^ contained in retarder should be responsible for this phenomenon, the complex calcium capacity of sulfonate groups is far weaker than carboxylic groups, when the pH value is 7, the carboxylic groups are still protonated and the anionic charge is mostly owed to the sulfonate groups presented in PAADM, apparently, PAADM are not subjected to chelation by calcium ions due to the weaken complex calcium capacity of sulfonate groups and the electrostatic repulsion between calcium ions and anionic N^+^. With the pH value increasing to 12, the anionic charge density of PAADM attains a significantly higher value of −463 C g^−1^. Addition of increased dosages of calcium ions to retarder solution decreases the anionic charge density to a final value of −360 C g^−1^ and gradually results in precipitation in this solution, as we see in Fig. 10, the retarder solution is gradually cloudy and produces white precipitates. It is because that at pH = 12 the carboxylic groups of the retarder have been deprotonated to make PAADM with more negative charges, and then the deprotonated carboxylic groups are easily complexed with calcium ions to produce precipitation in solution. It is also find from Fig. 10 that the final anionic charge density value (−360 C g^−1^) is obtained at a calcium concentration of 0.4 g L^−1^ and marks the saturation point for the uptake of Ca^2+^ by the carboxylate groups. According to this result, it is know that 1 g of PAADM can complex 4 g of Ca^2+^ ions, which indicate that PAADM possess strong calcium binding capacity. From the previous results, it is confirmed that PAADM possess obviously a very strong chelating ability to Ca^2+^ ions, which may play an important role in the retarding effect of the retarder on cement hydration.

#### Adsorption amount and zeta potential

**Fig. 11:**
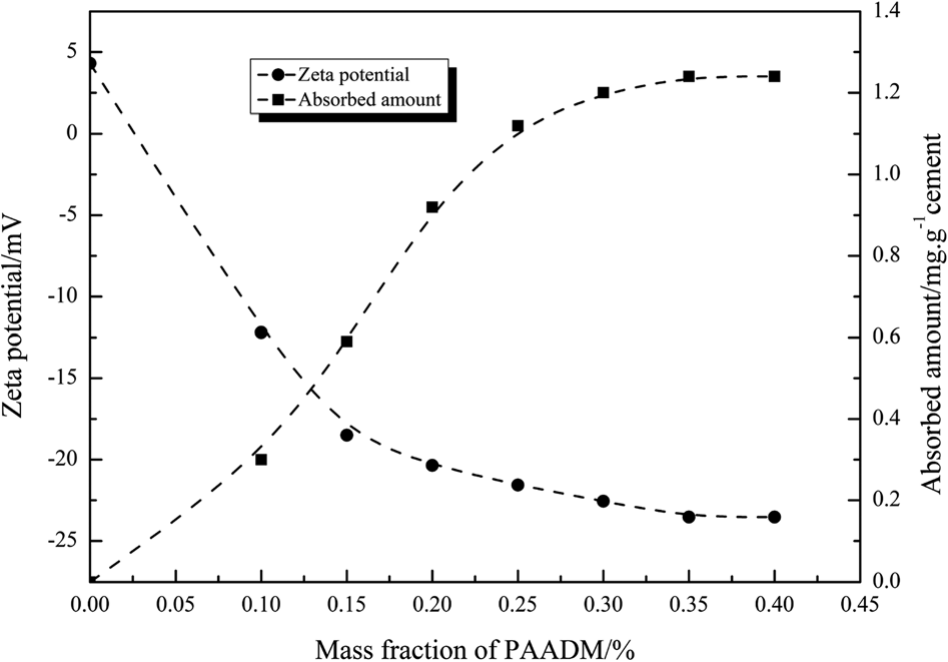
Absorbed amount of PAADM on oil well cement and zeta potential of oil well cement added with PAADM, measured at 25 °C.

The adsorption amount of PAADM on cement particles as well as the variation of zeta potential of setting cement slurries were presented in Fig. 11. As shown in Fig. 11, the adsorbed amount first increases quickly and then tends to be a stable state with mass fraction of retarder, where the adsorbed amount value is 1.26 mg g^−1^ and the mass fraction of retarder is 0.35%. It is clearly indicate that the retarder PAADM is easily adsorbed on the surface of cement particles. Meanwhile, the zeta potentials of the cement slurries occurs notable changes with the addition of retarder, the zeta potentials are remarkably decreased from 4.3 mV to a large negative value −23.53 mV at a retarder mass fraction of 0.35%. Thus, the results from Fig. 11 demonstrated that a negatively charged layer of polymer is formed on the surface of hydrating cement particles due to adsorption effect. This negatively charged layer not only inhibits the hydration process of cement attributed to the shielding effect of polymer layer, but also is favorable to the dispersion of cement particles because of electrostatic repulsion between the cement particles.^18–20^ The heterogeneous charge distribution on the surface of hydrating cement particles should be responsible for the adsorption behaviors between the cement particles and the retarders.^21–23^ As we all know, silicate phases possess a negatively charged surface whereas aluminate hydrates are usually positively charged when cement grains come into contact with water, at the same time, a large number of calcium ions is released from the surface of the cement particles into pore solution. On the one hand, because of the interaction of silicate phases anions with calcium ions, the silicate phases may possess partial positive charge properties. On the other hand, the reaction rate of aluminate phases is faster than that of silicate phases at the early stage. So a larger surface of fresh cement particles were covered with the positively charge, as is evidenced by a positive zeta potential value (4.8 mV). As a consequence, the cement particles provide more adsorption sites for the negatively charged polymers, PAAMD is be adsorbed on the negatively charged cement grains surface through electrostatic attraction forces, such way, the addition of PAADM decreases the zeta potential value of the cement pastes from 4.8 mV to a larger negative value −23.53 mV.

#### Phase analysis using XRD and SEM

**Fig. 12:**
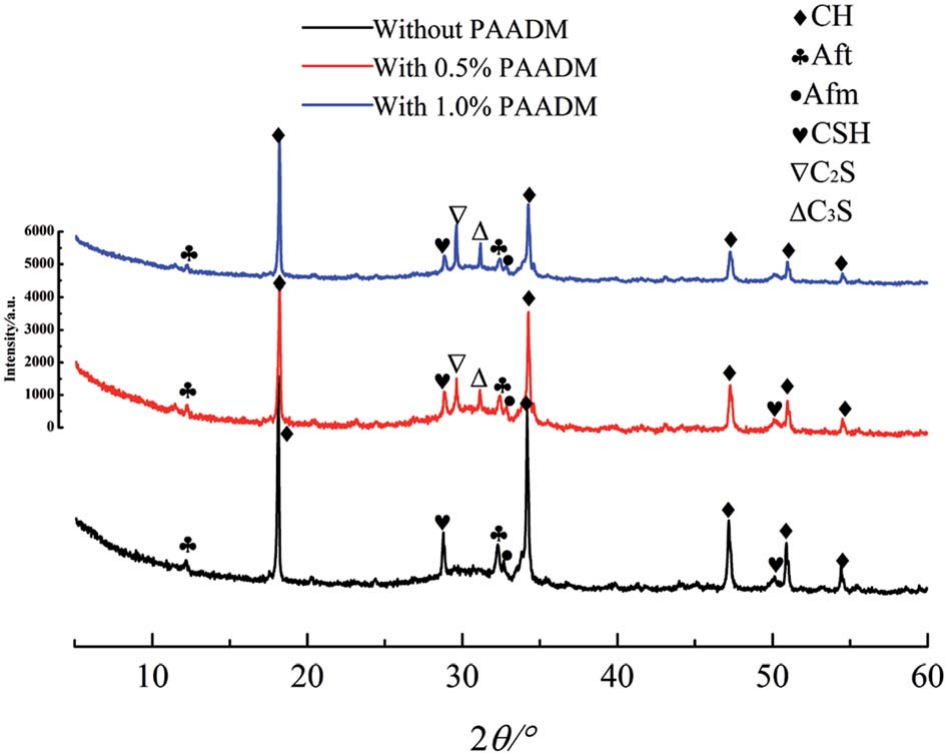
XRD patterns of cement slurry with or without retarder PAADM at curing conditions of 90 °C, 24 h.

**Fig. 13:**
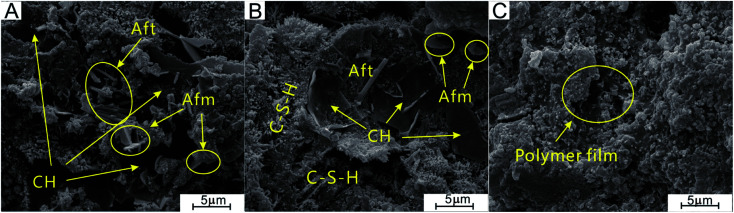
SEM photographs of set cement stone at curing conditions of 90 °C, 24 h. ((A) without PAADM; (B) and (C) different areas with 1.0% PAADM)).

The XRD patterns and the SEM photographs of setting cements cured at 90 °C for 24 h without or with different mass fraction of PAADM are displayed in Fig. 12 and 13 respectively. Combined with the inorganic crystal structure database and XRD patterns, as shown in Fig. 12, the hydration products of setting cement without PAADM are mainly composed of calcium hydroxide crystals (CH, 4.92, 2.628, 1.927 nm), ettringite crystals (AFt, 2.773, 2.616 nm), monosulfide hydrated calcium sulphoaluminate crystals (AFm, 2.74 nm) and hydrated calcium silicate gel (C–S–H, 3.35–3.12 nm).^24–26^ Compared with the cement without retarder, besides the aforementioned hydration products, the cement paste with retarder also appeared two calcium silicate (C_2_S) and three calcium silicate (C_3_S) phases, which originated from residues of unhydrated cement grains. At the same time, it can be observed that the diffraction peaks of calcium hydroxide crystals, ettringite crystals and monosulfide hydrated calcium sulphoaluminate crystals were weakened with the amount of retarder increased, and the diffraction peaks of the unhydrated cement grains (C_2_S and C_3_S) were enhanced. From the aforementioned analysis, it can be inferred that the retarder PAADM could inhibit the formation and growth of calcium hydroxide crystals, ettringite crystals and monosulfide hydrated calcium sulphoaluminate crystals, which leads to a delay the degree of cement hydration.

By comparing the SEM photographs of setting cements in Fig. 13, it can be seen from Fig. 13(A) that the hydration products of cement without PAADM include a large number of lamellar, sall flaky and needle columnar crystals, this above-mentioned crystals correspond to the hydration products Ca(OH)_2_, AFm and AFt. However, by observing the different region microstructure of set cement with 1.0% PAADM, it can been found that the aforementioned three crystal formations are significantly decreased (Fig. 13(B)), at the same time, a distinct layer of membrane is found to be covered on the surfaces of the hydrating cement particles (Fig. 13(C)), this phenomenon is in accord with the results of XRD analysis. From the aforementioned results, it can be deduced that the added retarder PAADM can form a polymer layer on the surfaces of the hydrating cement particles through complexation and adsorption effect, this adsorption layer could act as a isolation barrier between cement particles and water. Consequently, the hydration process of cement particles is delayed and the thickening time of cement slurry is prolonged.

#### Working mechanism of retarder PAADM

**Fig. 14:**
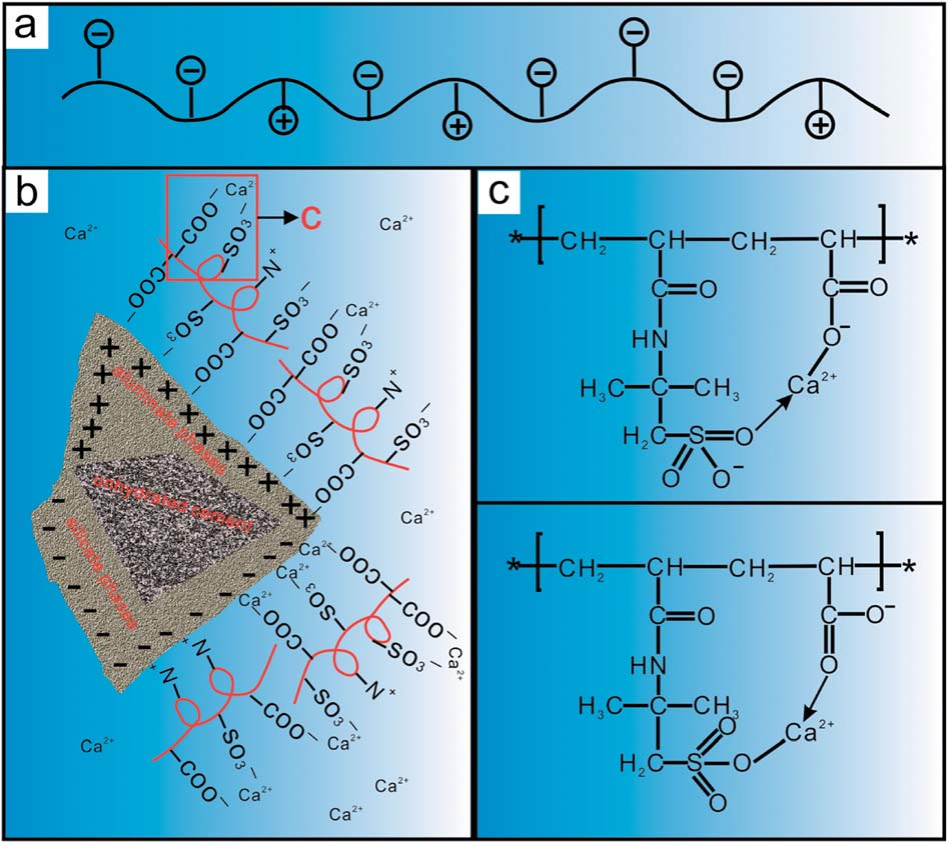
Schematic drawing of the molecular structure (a), the adsorption (b) and calcium complexation (c) of PAADM on the surface of cement particles.

Polymer retarders usually have different action forms with cement particles due to their diverse molecular structures and functional groups, which results in different retarding effects on the hydration of cement, so the retarding mechanism of many polymer retarders on cement are not generally understood and then worth further exploring. Combining the data gained from the calcium binding capacity, adsorption amount, zeta potential, the XRD and SEM analysis presented in this paper, the retarding mechanism of PAADM is proposed as follows:

It is known from Fig. 2 that the molecular structure of PAADM contains not only anion groups (carboxyl and sulfonate groups)but also cationic groups (quaternary ammonium groups), the schematic diagram of the molecular structure is shown in Fig. 14(a). As we all know, the surface of silicate phases (C_3_S and C_2_S) possess negatively charge whereas aluminate phases (C_3_A and C_4_AF) are usually positively charged. Under the effect of electrostatic force, the quaternary ammonium groups presented in retarder are adsorbed on the surface of the silicate phases, accordingly, the aluminate surfaces are wrapped by the carboxyl and sulfonate groups (Fig. 14(b)). When more and more retarder PAADM were gathered around the hydrated particles, a semipermeable polymer film playing the role of an isolating layer is formed gradually through adsorptive deposition effect. On the one hand, this isolation layer hinders the further contact between free water and cement particles, on the other hand, the diffusion rate of hydration ions, such as Ca^2+^, OH^−^, SiO_4_^4−^, into the solution is delayed by isolation layer. Therefore, the induction period of cement grains hydration is extended and the thickening time of cement slurry with PAADM is increased due to the above-mentioned two aspects of interaction.

Many literatures have shown that the oxygen atom has a strong coordination effect in the sulfonic and the carboxylic acid groups, especially the carboxylic acid group. As shown in Fig. 14(c), the C–O^−^ or S–O^−^ in the molecular structure of PAADM is firstly bonded with calcium ions, and then calcium ions are combined with S=O or C=O to form an irregular multiple chelating structure through complexation effect. In this way, the concentration of calcium ions in the cement slurry phase is greatly reduced, and the formation and development of calcium hydroxide crystals are prevented due to less calcium being available for their growth. As a result, the thickening time of cement slurry is prolonged effectively due to the calcium complexation effect. Based on the above analysis, it is reasonable that “adsorption deposition” and “calcium complexation” should be responsible for retarder PAADM delaying the hydration process of cement grains.

## Conclusion

(1) Characterization by Fourier transform infrared spectroscopy (FT-IR), X-ray diffraction (XRD) and thermogravimetric analysis (TGA) showed that the synthesized copolymer (PAADM) is the target product with intercalation/exfoliation composite structure and excellent thermal stability below 276.27 °C.

(2) The cement slurry containing PAADM has good retarding property in the range of 120–200 °C, and the thickening curve is smooth and stable, there are no abnormal phenomenon such as “bulging” and “walking steps”. Furthermore, the cement stone got rapid development of compressive strength under high temperature and low temperature conditions, this property could guarantee that the retarder PAADM is applied to the construction of deep wells and long interval wells.

(3) Based on the calcium binding capacity, adsorption amount, zeta potential, XRD and SEM analysis, it can be concluded that “adsorption deposition” and “calcium complexation” should be responsible for retarder delaying the hydration process of cement grains.

## Conflicts of interest

There are no conflicts to declare.

## Supplementary Material
